# Fret not thyself: The persuasive effect of anger expression and the role of perceived appropriateness

**DOI:** 10.1007/s11031-017-9661-3

**Published:** 2017-12-08

**Authors:** Jonathan Van’t Riet, Gabi Schaap, Mariska Kleemans

**Affiliations:** 1 Behavioural Science Institute, Radboud University, Nijmegen, The Netherlands; 2 Communication Science, Radboud University, PO Box 9104, 6500 HE Nijmegen, The Netherlands

**Keywords:** Anger, Emotion expression, Display rules, Persuasion

## Abstract

Anger expression is increasingly prevalent in Western mass media, particularly in messages that aim to persuade the audience of a certain point of view. There is a dearth of research, however, investigating whether expressing anger in mediated messages is indeed effective as a persuasive strategy. In the present research, the results of four experiments showed that expressing anger in a persuasive message was perceived as less socially appropriate than expressing non-emotional disagreement. There was also evidence that perceived appropriateness mediated a negative persuasive effect of anger expression (Study 2–4) and that anger expression resulted in perceptions of the persuasive source as unfriendly and incompetent (Studies 1 and 2). In all, the findings suggest that politicians and other public figures should be cautious in using anger as a persuasive instrument.

## Introduction

Around about the same time that Donald Trump famously declared “I will gladly accept the mantle of anger” (Hackman 2016), Senator Bernie Sanders proclaimed “Americans have a right to be angry” (Sanders 2016). As such, anger seems to have been one of the dominant themes of the United States 2016 presidential election. But being angry is not just the prerogative of American politicians; a strand of angry, populist politics has emerged in Europe well before Donald Trump and Senator Sanders (Koenis 2013). Nor is it confined to politicians; one study scrutinized data on 10 weeks of blogs, talk radio, and cable news and concluded that ‘outrage discourse’ is highly prevalent in American mass media (Sobieraj and Berry 2011).

There are likely many reasons for this. It has been noted that many people across the Western world are anxious about the future (Case and Deaton 2015; Krugman 2015), and angry at what they perceive as politicians’ disinterest and incompetence (Ford and Goodwin 2010). In a sense, then, anger expressed by politicians and media figures may simply be a reflection of widely felt anger among the population. In addition, anger may attract attention (Arpan and Nabi 2011; Calanchini et al. 2016). In a highly satiated media environment, forcefully expressed and emotive statements will likely receive higher ratings and higher click-through rates than relatively bland expressions of (dis)agreement (Mutz and Reeves 2005). But there may also be a third reason for anger expression in the media: it is possible that Mr. Trump, Senator Sanders, other politicians, as well as media figures more generally, express anger because they hope it will make them more persuasive.

There is strong evidence that expressing anger in interpersonal (non-mediated) communication can be an effective way to influence others (Sinaceur and Tiedens 2006; Van Kleef 2009; Van Kleef et al. 2004). Unfortunately, however, very little research has investigated whether these results generalize to the context of mediated messages (Van Kleef 2009). Although this seems plausible, it should be noted that anger expression as a persuasive strategy in mediated messages may violate accepted social norms for the polite expression of opinions (Mutz and Reeves 2005) and may therefore backfire as a persuasive strategy. To investigate which of these two outcomes is most likely, we conducted four experiments investigating the persuasive effects of anger expression in mediated messages.

## Anger in mediated messages

Anger generally arises from the perception that one’s concerns have been harmed or one’s progress towards goals obstructed, and it prepares for action to remove the obstacle (Frijda 2007). It is considered a negative emotion, resulting from an undesired state of affairs. Unlike other negative emotions, such as sadness, disgust, and fear, however, it is an activating emotion, resulting in approach rather than avoidance behavior (Potegal et al. 2010).

Most research on emotions as tools for social influence has focused on interpersonal communication (e.g., Elfenbein 2007; Van Kleef 2009). But emotional expression is not limited to situations in which there is direct interpersonal contact. Messages that are mediated through mass media or other channels can also contain emotional content and this too can influence recipients (Dillard and Peck 2001; Nabi 2010; Nabi and Wirth 2008). In particular, mediated messages can communicate the emotional expressions of others, as we have seen in the examples of President Trump and Senator Sanders.

Given the prevalence of mediated messages containing anger expression (Sobieraj and Berry 2011), it is surprising to see how little research has been done on its persuasive effects. Van Kleef and colleagues (2015) presented the results of five experiments showing that emotions expressed in mediated messages were able to influence recipients’ attitudes. Importantly, one of those studies (Study 4) investigated the effects of anger expression. It found that being exposed to a message containing anger directed at Greenpeace resulted in less positive attitudes towards Greenpeace than being exposed to a message containing happiness about Greenpeace. This lends support to the notion that emotion expression can have persuasive effects in the context of mediated messages. However, by contrasting expressions of anger with expressions of happiness, the expression of emotion in this study (happiness vs. anger) is confounded with the valence of the reaction (positive vs. negative). In the present research, we circumvented this problem by contrasting anger with (non-emotional) disagreement, so that only emotional expression differed between conditions, while (negative) valence is held constant.

## The social functional perspective on emotions

So how may anger expression affect the persuasive power of mediated messages? According to the social functional perspective on emotions (e.g., Elfenbein 2007; Van Kleef 2009), emotions contain valuable information about the feelings and intentions of the sender, which can have consequences for the behavior of the receiver. The ‘emotions as social influence’ (EASI) theory, developed by Van Kleef and colleagues (Van Kleef 2009; Van Kleef et al. 2011), explains how there are two ways in which emotion expression by the sender can influence the receiver (In the present paper, we refer to the person who expresses the emotion as ‘the sender’, while we refer to the person who observes the emotion expression as ‘the receiver’).

First, an inferential process can take place in which the receiver deduces information from the emotional expression. These inferences often take the form of ‘reverse appraisals’ (Hareli and Hess 2010), whereby receivers make inferences about the causes underlying the emotion, and adjust their own attitudes and behavior accordingly (Van Kleef et al. 2010). If for instance someone expresses anger about a colleague’s tardiness, this anger may lead the colleague to realize that being late is not acceptable, which may motivate punctuality in the future (Van Kleef et al. 2011).

The second way in which people may be influenced by others’ emotions is when receivers respond affectively rather than inferentially. When this happens, receivers form impressions about the person expressing the emotion. Expressions of positive emotions generally lead to increased liking, whereas expressions of negative emotions tend to lead to decreased liking (Clark and Taraban 1991). In the example of the angry colleague, the expressed anger may be upsetting and may lead to dislike. This constitutes an affective reaction, which in this case has unintended results, decreasing rapport between the two colleagues (Van Kleef et al. 2011).

So what determines whether people will react inferentially or affectively to expressions of anger? EASI theory stipulates that the receiver’s perception of the expressed emotion as socially acceptable is one important factor. High perceived acceptability will increase the likelihood of an inferential process, decrease the likelihood of affective reactions, and so foster social influence (Van Kleef et al. 2011).

Applied to the case of anger expression in mediated messages, there is the possibility that anger expression will trigger thoughts about the events that led to the anger, which may in turn help to persuade the receiver (inferential process). There is also the possibility, however, that anger expression is considered inappropriate, resulting not in persuasion, but in negative perceptions of the sender (affective reaction). As we will argue below, it is likely that questions of appropriateness loom larger for anger expression than for expressions of other emotions.

## The inappropriateness of anger

Research has shown that expressions of emotion are subject to socially learned norms. These norms are sometimes called ‘display rules’ (Ekman and Oster 1979), informing individuals whether and how to express emotions in social interactions. Because of the danger of anger expression for relationships (Averill 1982; Saarni and Von Salisch 1993; Von Salisch and Vogelgesang 2005), anger expression may be subject to especially strict display rules (Geddes and Callister 2007). In fact, most societies have developed strict social norms for the expression of anger. When the Psalmist admonishes us to ‘not fret ourselves’, this is not an isolated incident. Rather, both the Old and the New Testament are filled with warnings about the dangers of anger and resentment (Bateman and Jensen 1958). Similarly, the Buddha has famously declared that “holding on to anger is like drinking poison and expecting the other person to die”. Indeed, there is evidence that people regularly suppress anger in order to maintain healthy and successful social relationships (Ravid et al. 2010).

So what happens when the line for appropriate anger expression is crossed? Geddes and Callister (2007) argue that observers, rather than focus on the circumstances that caused the person to be angry, will be more likely to ascribe inappropriate anger to the person’s disposition (see also Kelley 1973). Using EASI theory’s terminology, an affective process will occur, and successful social influence is unlikely.

In sum, while a case can be made for the persuasive power of anger expression, there is also evidence to suggest that anger expression in the context of mediated messages will backfire when it is seen as socially inappropriate. The current research presents four experiments on the persuasive effect of anger expression in mediated messages to see which of these two outcomes is most likely.

In terms of hypotheses, we take a cue from the field of political communication. Although research in this area has mostly focused on ‘negative campaigning’ or ‘incivility’ rather than on anger expression per se, the results are informative. A study by Brooks and Geer (2007) found that messages containing incivility, which is usually seen as entailing a high degree of animosity (Funk 2001), were seen as less fair, less informative, and less important than civil messages. In fact, a ‘backlash’ effect of negative or uncivil messages is well-known in this literature (Fridkin and Kenney 2011). An explanation for this that has been put forward is the observation that expressing anger violates well-established social norms for the expression of opinions (cf. Mutz and Reeves 2005).

It could therefore be expected that anger expression is generally perceived as inappropriate in the context of persuasive mediated messages. We hypothesized that anger expression would lead to lower levels of perceived appropriateness and lower persuasion than expression of non-emotional disagreement. We also hypothesized that the negative effect of anger expression on persuasion would be mediated by perceived appropriateness.

It has been well established that people expect others to conform to social norms (Schachter 1951). Individuals engaging in deviant behavior can be expected to meet social disapproval or other, more severe, punitive measures (Chekroun and Brauer 2002). These expectations also extend to the domain of emotional expression (Szczurek et al. 2012). In a persuasive context, the likely result of flouting social norms is that the sender is perceived negatively, hindering his or her ability to change the receiver’s mind. Indeed, studies in advertising have shown that ‘offensive’ advertising, i.e. advertising containing rude language, violence, explicit sexual themes, or content violating social norms in some other way (Beard 2008; Prendergast et al. 2008), results in negative perceptions of the advertising (Ketelaar et al. 2012) and the advertised product (Chan et al. 2007). As such, it can be expected that, if expressions of anger are perceived as inappropriate, they will result in lower levels of persuasion than more appropriate expressions of non-emotional disagreement.

It should be stressed that we do not expect perceived appropriateness to be a sufficient condition for persuasion. Successful persuasion will not occur simply because a message is communicated in a socially appropriate way. Rather, we expect that perceived inappropriateness reduces the likelihood of successful persuasion: persuasive messages will be less successful to the extent that they are perceived as inappropriate.

We also investigated the effects of anger expression on perceptions of the sender as competent and likable in Study 1 and 2. If an affective process results in negative perceptions of the sender, there should be lower perceived likability and competence scores in the anger expression condition than in the non-emotional disagreement condition. Previous research has already shown that anger expression can lead to perceptions that the sender is unfriendly and cold (Kim and Niederdeppe 2014; Tiedens 2001). Findings for perceived competence are mixed, however (Kim and Niederdeppe 2014; Tiedens 2001). It is therefore important to shed more light on the issue of how anger expression affects perceptions of likability and, especially, competence.

## The present research

In the present research, we exposed participants to fabricated news articles in which a decision by a public institution was announced. In these articles, a person was quoted making a persuasive argument against the decision (hereafter referred to as ‘the sender’). The news articles were manipulated such that this person expressed different levels of anger. In line with previous research (Calanchini et al. 2016), participants’ agreement with the sender’s position was assessed as the main outcome measure. In the case of a successful inferential process, we would expect persuasion to be higher in the anger condition than in the disagreement condition. As detailed above, however, we expected anger expression to be perceived as inappropriate and to result in negative perceptions of the sender. In other words, we reasoned that an affective process would be more likely, ultimately leading to lower persuasion in the anger condition than in the disagreement condition. Therefore, we assessed perceived appropriateness and persuasion in Studies 1–4, as well as participants’ perceptions of the sender as competent and likable in Study 1 and 2. The complete stimulus materials for Studies 1–4, as well as all questions and the resulting data, can be found at https://osf.io/nzxma/.

## Study 1

### Method

#### Design and procedure

Study 1 had a one-factorial design with three conditions: anger expression versus mild anger expression versus expression of disagreement. Thus, three versions of the same news article were created. Each participant was randomly assigned to one of these newspaper articles. Participants participated in exchange for course credit. They took part in the study in groups of approximately 15 persons at the same time but were individually assigned to conditions. They received a print booklet that contained all materials and measures and were asked to fill in the questionnaires using pens or pencils. An experimenter was present to provide a short introduction of the study. Participants first answered questions on demographics, and then read the fabricated news article. After reading it, participants answered a number of questions about the article. After the study, participants were debriefed and thanked for their participation.

#### Participants

A total number of 67 undergraduate students (28.4% male) participated in Study 1. Their age ranged between 17 and 22 years old (*M* = 18.72; *SD* = 0.97). There were no significant differences between conditions in terms of gender, χ^2^(2) = 3.08, *p* = .21, or age, *F*(2, 64) = 0.88, *p* = .88, η_p_^2^ = .00.

#### Stimulus materials

For the fabricated news article, we chose the same topic as was used in a previous study by Van Kleef et al. (2015, Study 2). We created a news article announcing the cancellation of a popular quiz show ‘Lingo’ broadcasted by the Dutch public television network. This scenario was based on events in 2006, when plans emerged to cancel the program, causing a commotion in the Netherlands (Trouw 2006) that resulted in continuation of the program. The article that is used in the current study is based on news messages that reported on this incident. However, as Study 1 was conducted in 2013, the fabricated news article stated that there had been a recent revival of the plan to cancel Lingo. A ‘media expert’ (i.e., the ‘sender’) was quoted commenting on these plans.

A notable difference between our study and the one conducted by Van Kleef and colleagues (2015) is that the latter manipulated the message to communicate either sadness or joy, or no social information, while we were interested in the effects of anger. As mentioned above, previous research on the effects of emotion expression has mostly manipulated the expressed emotion to be either positive or negative. For instance, Tiedens (2001) contrasted expressions of sadness with expressions of anger and Van Kleef et al. (2015, Study 4) contrasted anger with joy. This makes sense if we have reason to expect opposing effects in these conditions, and want to establish whether there is an overall effect of emotion expression. But if we want to obtain an estimate of the effect of anger per se, irrespective of the effects of joy, sadness or any other emotion, it makes more sense to contrast an anger condition with an emotionally neutral condition.

Furthermore, we argue that it is necessary that the sender in this emotionally neutral condition expresses disagreement. Previous studies have used control conditions in which the sender does not express any opinion on the subject matter, but merely ‘observes’, or ‘noted’ ongoing events (Van Kleef et al. 2015, Study 2). But this confounds emotional expression with the valence (positive or negative) of the reaction. After all, if the sender only ‘observes’ what is going on without commenting, we can deduce that he or she has no opinion on the matter. If the sender is angry, however, it is clear that he or she disagrees with what is going on. Therefore, contrasting a sender who is angry with a sender who disagrees allows for a more accurate assessment of the effect of emotional expression over and above the possible effect of non-emotional social influence. Consequently, we manipulated the message so that the sender was either angry or disagreed with the decision to cancel the TV show in question. Such verbal manipulations of emotional expressions have been successfully employed in previous research (Van Kleef et al. 2015).

In all conditions, the sender made a persuasive argument against the decision. To manipulate anger expression, the sender was described as either ‘furious’ about the decision (anger condition), ‘annoyed’ about the decision (mild anger condition) or as ‘not in agreement’ with the decision (non-emotional disagreement condition), calling it either ‘an outrage’, ‘unfair’ or ‘unwise’ in the respective three conditions. The arguments that were used to support his opinion did not differ between conditions. In all conditions, the sender supported his opinion with the argument that Lingo has been on Dutch television for 25 years, and with the position that it is the task of public service broadcasting to serve audiences irrespective of commercial interests. Each version of the article ended with a conclusion that more information about the decision will be announced soon.

As anger mostly arises from a perceived harm of concerns (Frijda 2007), it is not uncommon for people to be angry when something is taken away from them. In this light, it is notable that, in 2006, one Member of Parliament indicated that he was ‘furious’ about the plan to cancel Lingo, angrily demanding an explanation from the Secretary of Culture (Trouw 2006).

#### Outcome measures

##### Perceived appropriateness

Perceived appropriateness was measured with one item, asking participants to indicate how appropriate they thought the sender’s contribution was (*M* = 5.16; *SD* = 1.18).

##### Persuasion

Following previous research (Calanchini et al. 2016), we assessed participants’ agreement with the decision to cancel the TV show. On a 7-point scale, ranging from 1 (totally disagree) to 7 (totally agree), participants indicated to what extent they agreed with the items ‘Lingo must stay’, ‘It is a bad idea to cancel Lingo’, ‘There are good reasons to stop airing Lingo’, and ‘The network is right to cancel Lingo’. The scores on the last two items were reversed, so that higher scores indicated increased agreement with the sender’s persuasive appeal (see Calanchini et al. 2016). All items were averaged into an attitude index (*M* = 5.09; *SD* = 1.05; Cronbach’s α = .77).

##### Perceived likability and perceived competence

We assessed participants’ ratings of likability and competence with regards to the sender. In line with previous research (Kim and Niederdeppe 2014), *liking* was assessed with four items, in which participants were asked to indicate on a 7-point scale how warm, nice, kind, and friendly they perceived the sender to be. Scores on the items were reversed so that a higher score indicated more liking. A mean score of the four items was calculated (*M* = 4.29; *SD* = 1.15, α = .91).

In line with previous research (Fridkin and Kenney 2011), *competence* was assessed with ten items that asked participants to use a 7-point scale (1 = *very much*; 7 = *not at all*) to indicate how rational, sensible, intelligent, calculating, good at his job, knowledgeable, down-to-earth, professional, business-like, and no-nonsense they perceived the sender to be. Scores were reversed before a mean score was calculated, such that higher scores indicate greater perceived competence (*M* = 4.16; *SD* = 0.95; α = .89).

#### Data analysis

Analyses of variance (ANOVAs) were performed with anger condition as the independent variable and perceived appropriateness, persuasion, and likability and competence as the dependent variables. No covariates were included in the models. Partial eta squared was used as the effect size measure for the F-test. Hedges’ g was used as the effect size measure for two-way comparisons. Besides the variables mentioned in this report, a limited number of additional variables was assessed in Study 1 for exploratory purposes. These did not affect the results, and are not of central concern to the present paper.

### Results

#### Perceived appropriateness

The analyses revealed a significant effect of the anger manipulation on perceived appropriateness, *F*(2, 64) = 12.72, *p* < .001, η_p_^2^ = .28. Post-hoc tests showed that perceived appropriateness was significantly lower in the anger condition than in the mild anger condition, *p* < .01, Hedges’ *g* = − 0.88, and significantly lower in the mild anger condition than in the disagreement condition, *p* < .01, Hedges’ *g* = − 0.64 (see Table 1).

**Table 1:** Means and standard deviations of outcome measures in Study 1

	Anger		Mild anger		Disagreement	
	M	SD	M	SD	M	SD
Perceived appropriateness	4.32^a^	1.43	5.35^b^	0.78	5.82^c^	0.66
Persuasion	4.99	1.12	5.02	0.93	5.25	1.12
Likability	3.58^a^	1.23	4.61^b^	0.99	4.66^b^	0.92
Competence	3.48^a^	0.94	4.48^b^	0.63	4.50^b^	0.90

Different superscripts within a row indicate significant differences between conditions at *p* < .05

#### Persuasion

With regards to persuasion, there was no significant main effect of the anger manipulation, *F*(2, 64) = 0.40, *p* = .67, η_p_^2^ = .01. The contrast between the anger condition and the disagreement condition was *g* = − 0.23.

#### Likability and competence

The analyses revealed that the anger manipulation affected participants’ perceptions of the sender. With regards to perceived likability, there was a main effect of the anger manipulation, *F*(2, 64) = 7.42, *p* < .01, η_p_^2^ = .19. Post-hoc tests showed that the angry sender was perceived as less likable than the mildly angry sender, *p* < .01, *g* = 0.91, and the sender expressing disagreement, *p* = .03, *g* = 0.98, with no difference between the sender expressing disagreement and the mildly angry sender, *p* = .51, *g* = 0.05.

With regards to competence, there was a main effect of the anger manipulation, *F*(2, 64) = 10.92, *p* < .001, η_p_^2^ = .25. Post-hoc tests revealed that the angry sender was perceived as less competent than the mildly angry sender, *p* < .001, *g* = 1.23, and the sender expressing disagreement, *p* < .001, *g* = 1.09, with no difference between the mildly angry sender and the sender expressing disagreement, *p* = .98, *g* = 0.03.

#### Mediation

Finding a strong effect of anger expression on perceptions of appropriateness, we investigated whether perceived appropriate would mediate an effect of anger expression on persuasion. Although the total effect of anger expression on persuasion was not significant, it is still possible that there is an indirect effect of anger expression on persuasion through perceived appropriateness (Hayes 2009).

To investigate this possibility, we used a dummy variable that contrasted the anger conditions with the disagreement condition (dummy-coded 0 = disagreement; 1 = anger), ignoring the mild anger condition. We used Hayes’ (2012) PROCESS macro for SPSS to formally assess the indirect effect of anger expression on persuasion through perceived appropriateness. As described above, anger versus disagreement had a significant effect on perceived appropriateness (path a). The results of an analysis with condition and perceived appropriateness as the independent variables and persuasion as the dependent variable showed that perceived appropriateness did not significantly affect persuasion, *B* = 0.09, *p* = .58, bootstrapped CI [− 0.23 to 0.40] (path b). The indirect effect (path a × path b) was ab = − 0.13, bootstrapped CI [− 0.67 to 0.36], with an associated effect of κ^2^ = .05.

## Discussion and introduction to Study 2

The results of Study 1 revealed that anger expression is considered less appropriate than expression of disagreement. A non-significant effect was found for persuasion, but significant and strong effects were found for perceived likability and competence: anger expression resulted in perceptions of the sender as low in likability and competence. All in all, the results do not seem to lend support for the notion that anger expression is persuasive. Instead, anger expression seems to be considered inappropriate and seems to lead to negative perceptions of the sender.

In Study 1, perceived appropriateness only had a small effect on persuasion, resulting in small-to-medium sized mediation. Perhaps the failure to find stronger evidence for mediation was due to our use of single-item measure for perceived appropriateness. Therefore, we used a composite scale to assess perceived appropriateness in Study 2. In addition, we added a neutral control condition to the design, in which the sender indicated to ‘have heard’ about the decision, but did not himself offer an opinion on the issue.

## Study 2

### Method

#### Design and procedure

The study employed a one-factorial (anger condition: neutral versus disagreement vs. mild anger vs. anger) between-subjects design. Undergraduate students participating in the study in exchange for course credits were randomized into the four conditions. Participants were invited into our lab and seated in individual soundproof booths where they took part in the study. Desktop computers were used to present all materials.

In a procedure similar to the procedure of Study 1, participants first answered questions on demographics, and then read the fabricated news article. After reading it, participants answered a number of questions about the article. At the end of the study, participants were debriefed and thanked for their participation.

#### Participants

A total number of 148 undergraduate students (23.0% male) participated in Study 2, with age ranging from 18 to 51 (*M* = 22.27; *SD* = 4.45). There were no significant differences between conditions in terms of gender, χ^2^(3) = 1.63, *p* = .65, or age, *F*(3,143) = 0.56, *p* = .64, η_p_^2^ = .01.

#### Stimulus materials

We used the same topic as described in Study 1 to create the stimulus materials. Plans to cancel Lingo were mentioned, after which comments by a ‘media expert’ (the sender) were quoted. The sender supported his opinion with the same arguments as in Study 1. In the neutral version of our article, the text stated that the sender ‘had heard’ about the decision to cancel Lingo, after which he explained that the Dutch public television network had had much hesitation before cancelling the program, citing the two arguments also used in Study 1. Importantly, the sender did not express his personal opinion in this condition. The other conditions employed the same materials as in Study 1.

#### Outcome measures

##### Perceived appropriateness

Perceived appropriateness was assessed with four items asking participants to indicate the extent to which they thought the sender’s response was appropriate, the extent to which they thought the response fitted the topic at hand, the extent to which they thought the response was fitting for a news article, and the extent to which they thought the response was understandable, all on a 7-point scale (1 = *not at all*; 7 = *very much*). These four items were averaged into a single appropriateness scale (*M* = 5.20; *SD* = 0.89; α = .74).

##### Persuasion

Participants’ agreement with the sender’s position was assessed with the same four items as in Study 1 (*M* = 5.75; *SD* = 2.91, α = .76) where higher scores indicated increased agreement with the sender’s persuasive appeal (see Calanchini et al. 2016).

##### Perceived likability and perceived competence

Perceived likability (*M* = 4.15; *SD* = 1.17; α = .90) was assessed with the same items that were used in Study 1, as was perceived competence (*M* = 4.01; *SD* = 0.81, α = .85).

#### Data analysis

Procedures for data analysis were equal to those in Study 1. As in Study 1, a limited number of additional variables were assessed for exploratory purposes. These did not affect the results, and are not of central concern to the present paper.

### Results

#### Perceived appropriateness

The analyses for perceived appropriateness revealed a significant main effect of condition, *F*(3, 142) = 2.90, *p* = .04, η_p_^2^ = .06. Further investigation of the means revealed that perceptions of appropriateness were highest in the disagreement and mild anger condition and lowest in the neutral and anger condition (see Table 2). The difference between the disagreement and anger condition was significant, *p* = .02, *g* = − 0.46.

**Table 2:** Means and standard deviations of outcome measures in Study 2

	Anger		Mild anger		Disagreement		Neutral	
	M	SD	M	SD	M	SD	M	SD
Perceived appropriateness	4.98^a^	0.91	5.42^b^	0.86	5.39^b^	0.87	4.98^a^	0.86
Persuasion	4.82	0.93	5.22	1.07	5.11	0.87	5.20	1.09
Likability	3.90^a^	1.10	4.38^b^	1.10	4.44^b^	1.38	3.86^a^	1.01
Competence	3.61^a^	0.75	3.90^ab^	0.94	4.19^b^	0.70	4.36^b^	0.65

Different superscripts within a row indicate significant differences between conditions at *p* < .05

#### Persuasion

With regard to persuasion, the results showed that the effect of the anger manipulation was far from significant, *F*(3, 144) = 1.31, *p* = .27, η_p_^2^ = .03. The difference between the anger and disagreement condition was characterized by a small to medium-sized effect, *g* = − 0.32, *p* = .17.

#### Likability and competence

The analyses revealed that condition affected participants’ perceptions of the sender. With regard to perceived likability, there was a marginally significant main effect of condition, *F*(3, 144) = 2.59, *p* = .06, η_p_^2^ = .05, with the neutral sender and the angry sender receiving particularly low likability scores. The anger condition received significantly lower likability scores than the disagreement, *p* = .02, *g* = 0.43, and the mild anger condition, *p* = .01, *g* = 0.43.

With regard to competence, there was a main effect of the anger manipulation, *F*(3, 142) = 6.71, *p* < .001, η_p_^2^ = .12. The angry sender was perceived as significantly less competent than the sender expressing disagreement, *p* < .01, *g* = 0.79, and also than the neutral sender, *p* < .01, *g* = 1.06. Perceptions of the mildly angry sender did not differ significantly from perceptions of the other senders, ps > .10, 0.33 < *g*s < 0.56.

#### Mediation

In our mediation analysis, we used a dummy variable that contrasted the anger condition with the disagreement condition (dummy-coded 0 = disagreement; 1 = anger). The results of the ANOVA showed that anger versus disagreement had a significant effect on perceived appropriateness (path a). The results of the second analysis, with condition and perceived appropriateness as the independent variables and persuasion as the dependent variable, showed that perceived appropriateness significantly affected persuasion, *B* = 0.25, *p* = .04, bootstrapped CI [0.01–0.48] (path b). The indirect effect (path a × path b) was also significant, ab = − 0.10, bootstrapped CI [− 0.32–0.00], with an associated effect size in the small-to-medium range, κ^2^ = .06, suggesting substantial mediation by perceived appropriateness.

## Discussion of Study 2 and introduction to Study 3

The results of Study 2 confirmed the finding from Study 1 that anger expression is considered less appropriate than expression of disagreement. In contrast to Study 1, however, the results of Study 2 revealed evidence for an indirect effect of anger expression on persuasion through perceived appropriateness. In addition, anger expression led to lower levels of perceived likability and competence. All this supports the notion that anger expressions flouts ‘display rules’, is therefore considered inappropriate, and is ineffective as a persuasive strategy. It is possible, however, that the negative persuasive effect of anger expression was at least partly a consequence of the specific topic discussed in our stimulus materials. Although news media featured rather heated discussions about whether or not to cancel Lingo in 2006 (Trouw 2006), triggering Van Kleef et al. (2015) to use this topic in their research, the supposed return of these plans may have seemed of little relevance to our undergraduate sample. On the other hand, participants’ scores on our persuasion measure were on average well above the mid-point of the scale, indicating that they generally supported the sender’s position. Nevertheless, to increase confidence in the generalizability of the effects, we performed a third study, using a different topic. To increase the perceived relevance of the topic, we employed undergraduates as the participants and messages about University policy as the stimulus materials.

While perceived appropriateness mediated a persuasive effect in Study 2, the total effect of anger expression on persuasion was not significant. Potentially, other mediators are at work, facilitating a positive indirect effect of anger expression, that effectively cancel out the negative indirect effect of perceived appropriateness. In Study 3, we investigated whether participants’ experienced anger about the policy proposals would facilitate such a positive indirect effect. Research shows a consistent correlation between self-reported anger about perceived injustices on the one hand and the willingness to act to remedy these injustices on the other hand (Van Doorn et al. 2014). It is therefore of interest to assess whether anger expression by a sender can result in increased levels of anger as experienced by the receiver, and whether increased levels of anger can contribute to persuasion.

## Study 3

### Method

#### Design and procedure

The study employed a 2 (topic) × 2 (anger condition: disagreement vs. anger) between-subjects design. Undergraduate students participating in the study in exchange for course credits were randomized across the four conditions. Participants were invited into our lab and seated in individual soundproof booths where they took part in the study. They received a print booklet that contained all materials and measures and were asked to fill in the questionnaires using pens or pencils. The procedure was identical to Study 1 and 2.

#### Participants

A total number of 95 undergraduate students (11.6% male) participated in Study 3, with age ranging from 17 to 29 (*M* = 19.33; *SD* = 2.11). There were no significant differences between conditions in terms of gender, χ^2^(3) = 1.15, *p* = .77, or age, *F*(3,91) = 0.65, *p* = .58, η_p_^2^ = .02.

#### Stimulus materials

In early 2015, there was widespread discussion in Dutch academia about higher education policy. At the University of Amsterdam, activist students even occupied administrative offices as a protest against cost-cutting measures, demanding increased student participation in decision making (NRC 2015). We used this controversy as a background for our study, creating fabricated news articles that introduced measures ‘to improve teaching’, which would allegedly be introduced at our university.

To increase confidence that the obtained effects can be generalized across contexts, we created two messages, dealing with different topics. In one condition, the news article mentioned a plan to increase the number and/or duration of classes. At our university, policy demands that undergraduate students have at least 15 h of scheduled classes per week. This policy is widely unpopular among students, who prefer high quality of teaching, rather than quantity. It was stated in the news article that the university board had plans to change the ‘15-h rule’ into an ‘18-h rule’, thereby increasing the quantity of teaching. In the second condition, the news article mentioned a plan to abolish study programs that attract only limited numbers of students. Such plans were actually discussed at a number of Dutch universities in the spring of 2015 as a means to cut costs, but met with widespread resistance from students and faculty.

In a pre-test among 59 undergraduates from the same participant pool, we asked participants to indicate their agreement with the two proposals on a 7-point scale (1 = *totally disagree*; 7 = *totally agree*). The results showed that participants generally disagreed with both proposals, as indicated by an average score below the midpoint (*M*_small−programs_ = 3.31; *SD*_small−programs_ = 1.60; *M*_18-h_ = 3.73; *SD*_18-h_ = 1.44). Participants also rated the 18-h plan as having a significantly greater effect on their lives (*M* = 5.19; *SD* = 1.17) than the small-programs plan (*M* = 2.20; *SD* = 1.23), *t*(58) = 12.37, *p* < .001, *g* = 2.67, on a 7-point scale, ranging from 1 = *very small effect* to 7 = *very large effect*.

#### Outcome measures

##### Perceived appropriateness

Perceived appropriateness was assessed with the same four items as in Study 2, which were averaged into a single appropriateness scale (*M* = 4.65; *SD* = 1.15; α = .79).

##### Persuasion

Participants’ agreement with the sender’s position was assessed with the same four items as in Study 1 and 2 (*M* = 4.74; *SD* = 1.13, α = .85) where higher scores indicated increased agreement with the sender’s persuasive appeal (see Calanchini et al. 2016).

##### Self-reported anger

Following previous research (Glasford 2013), we asked participants to indicate the extent to which they agreed with the statements ‘I am annoyed that the university board considers [the proposal]’ and ‘I get really worked up about [the proposal]’ on a 7-point scale (1 = *I don’t agree at all*; 7 = *I totally agree*). These two items were significantly correlated, *r* = .67, *p* < .001.

#### Data analysis

Data analysis was equal to Study 1 and 2, with the exception that a potential interaction between topic and anger condition was also investigated. As in Study 1 and 2, a limited number of additional variables were assessed for exploratory purposes. These did not affect the results, and are not of central concern to the present paper.

### Results

#### Perceived appropriateness

The analyses for perceived appropriateness revealed a significant main effect of the anger manipulation, *F*(1, 91) = 7.04, *p* < .01, η_p_^2^ = .07. Further investigation revealed that perceptions of appropriateness were higher in the disagreement condition than in the anger condition, *p* < .01, *g* = 0.50. There was also a significant main effect of topic, *F*(1, 91) = 19.74, *p* < .001, η_p_^2^ = .18, with participants deeming the source’s reactions more appropriate in the ‘small programs plan’ condition than in the ‘18-h plan’ condition, *p* < .01, *g* = 0.88. There was no significant interaction between topic and anger, *F*(1, 91) = 0.01, *p* = .93, η_p_^2^ = .00 (see Table 3).

**Table 3:** Means and standard deviations of outcome measures in Study 3

	Anger		Disagreement	
	M	SD	M	SD
Perceived appropriateness	4.36^a^	1.25	4.93^b^	0.98
Persuasion	4.65	1.18	4.84	1.08
Self-reported anger	2.80	1.52	2.79	1.39

Different superscripts within a row indicate significant differences between conditions at *p* < .05

#### Persuasion

With regards to persuasion, the results showed that there was no significant main effect of the anger manipulation, *F*(1, 89) = 0.64, *p* = .43, η_p_^2^ = .01. The contrast between the anger condition and the disagreement condition was *g* = − 0.17. There was a significant main effect of topic, *F*(1, 89) = 9.12, *p* < .01, η_p_^2^ = .09. Further investigation revealed that participants showed higher persuasion in the ‘small programs plan’ condition (*M* = 5.08; *SD* = 0.95), than in the ‘18-h plan’ condition (*M* = 4.40; *SD* = 1.20), *p* < .01, *g* = 0.62. There was no significant interaction between topic and anger, *F*(1, 89) = 0.56, *p* = .46, η_p_^2^ = .01.

#### Self-reported anger

The analyses for self-reported anger revealed no significant effects of the anger manipulation, *F*(1, 88) = 0.00, *p* = .96, η_p_^2^ = .00, topic, *F*(1, 88) = 0.25, *p* = .62, η_p_^2^ = .00, or the interaction between anger and topic, *F*(1, 88) = 0.00, *p* = .99, η_p_^2^ = .00.

#### Mediation

The results of the ANOVAs showed that perceived appropriateness qualified as a potential mediator, but self-reported anger did not. For the mediation analysis with perceived appropriateness, we used a dummy variable (coded 0 = *disagreement*; 1 = *anger*), collapsing the data across topic condition. The results of the ANOVA had shown that anger versus disagreement had a significant effect on perceived appropriateness (path a). The results of the second analysis, with condition and perceived appropriateness as the independent variables and persuasion as the dependent variable, showed that perceived appropriateness significantly affected persuasion, *B* = 0.45, *p* < .001, bootstrapped CI [0.27–0.65] (path b). The indirect effect (path a × path b) was also significant, ab = − 0.28, bootstrapped CI [− 0.54 to − 0.06], κ^2^ = .13, suggesting substantial mediation by perceived appropriateness.

## Discussion of Study 3 and introduction to Study 4

The results of Study 3 confirmed the finding from Study 1 and 2 that anger expression is considered less appropriate than expression of disagreement. In addition, there was evidence for an indirect effect of anger expression on persuasion through perceived appropriateness. The topic used in Study 3 was arguably more relevant for our participants than in Study 1 and 2, increasing our confidence in the revealed effects. To increase our confidence even further, however, we conducted a fourth study, choosing a dispute about the ‘Transatlantic Trade and Investment Partnership’ (TTIP) as our third topic.

Study 3 also found that anger expression did not affect participants’ self-reported anger. However, as in Study 1 and 2, Study 3 revealed a non-significant total effect of anger expression on persuasion. So there is still the possibility of a separate psychological process, affecting persuasion in a positive way and counteracting the negative effects of anger expression via perceived inappropriateness. One potential candidate could be perceptions of the sender as dominant, high status, and/or an effective leader. Dominant people and those with high social status are known to exhibit anger more frequently than submissive people and those with low social status (Tiedens et al. 2000). As a result of this, people often attribute dominance and high status to those who express anger (Tiedens et al. 2000). In Study 4, we therefore investigated whether perceptions of the sender as dominant and high status would qualify as potential mediators of a positive persuasive effect of anger expression.

## Study 4

### Method

#### Design and procedure

Study 4 had a one-factorial design with two conditions: anger expression versus expression of non-emotional disagreement. Each participant was randomly assigned to read one of two news articles that were created. Participants were invited into our lab and seated in individual soundproof booths where they took part in the study, in exchange for €7.50. They completed all materials and measures on a desktop computer. Participants first answered questions on demographics and were then exposed to a fabricated news article featuring a dispute about TTIP in which a ‘sender’ was quoted who either voiced disagreement with TTIP or expressed anger about TTIP. The sender supported his opinion with the same argument in all conditions. Subsequently, the post-test questionnaire assessed the dependent variables of the study. After taking part in the study, participants were thanked and debriefed.

#### Participants

A total number of 96 undergraduate students at a Dutch university (22.9% male) participated in Study 4. Their age ranged between 18 and 63 years old (*M* = 23.48; *SD* = 6.46). There were no significant differences between conditions in terms of gender, χ^2^(1) = 0.24, *p* = .63, age, *t*(94) = − 1.20, *p* = .23.

#### Stimulus materials

Study 4 used a dispute about the TTIP as the topic of the persuasive messages. TTIP was chosen as a context for our manipulations because the topic of free trade elicits strong opinions and angry reactions from many people, both in mass and social media (Euronews 2016). It has been an important rallying cry for Donald Trump in his 2016 presidential campaign, where he has frequently combined opposition with free trade with expressions of anger (Broad 2017). In the Netherlands TTIP specifically has frequently been the target of public anger (AD 2016). Thus, TTIP constituted an ecological valid high relevance topic.

In the fabricated news article, a (fictional) Danish member of the European Parliament (hereafter called ‘the sender’) was quoted as either disagreeing with TTIP in non-emotional terms (disagreement condition) or as being angry about it (anger condition). The sender was described as either “furious” or as “not in agreement”. He called TTIP either “a disgrace” or “not a good idea”, and argued that TTIP should be “thrown in the rubbish bin” or that it “should not be signed”. In all conditions, the sender supported his opinion with the argument that free trade is bad for workers, as they have to compete with workers abroad.

#### Outcome measures

##### Perceived appropriateness

Perceived appropriateness was assessed with the same four items as in Study 2 and 3, which were averaged into a single appropriateness scale (*M* = 4.84; *SD* = 0.88; α = .69).

##### Persuasion

Participants’ agreement with the sender’s position was assessed with the same four items as in Study 1–3 (*M* = 3.60; *SD* = 1.03, α = .83).

##### Perceived dominance

Following previous research (Hareli et al. 2009), we distinguished between perceived dominance and perceived submissiveness as two potentially separate judgments. Perceived dominance was assessed with three items asking participants to indicate the extent to which they thought the sender seemed like a leader type, the extent to which the sender seems like someone who gives orders that are obeyed, and the extent to which the sender seemed like someone who insists on things. Perceived submissiveness was assessed with three items asking participants to indicate the extent to which they thought the sender seemed obedient, the extent to which the sender seems like someone who accepts orders and obeys them, and the extent to which the sender seemed like someone who does not insist on things (Hareli et al. 2009, Study 2). A factor analysis revealed no clear support for the expected two-factor solution, however. Instead, the items of the perceived submissiveness scale loaded on the same dimension as the third dominance item, while the other two dominance items were only weakly correlated. Therefore, we used the perceived submissiveness items (reverse coded) and the third dominance item to construct our measure of perceived dominance (*M* = 5.33; *SD* = 0.84, α = .76).

In addition, status conferral was assessed with four items based on procedures that were developed by Tiedens and colleagues (2000). We asked participants how much experience they though the sender had in politics, how successful they thought the sender had been in his career in politics, how successful they thought the sender would be in his future career, and whether they would hire the sender if they were the CEO of a large company and the sender would apply for a relevant position (*M* = 4.69; *SD* = 0.89, α = .82).

#### Data analyses

We used *t* tests to investigate differences in means between conditions for perceived appropriateness, persuasion, perceived dominance, and status. Hedges’ g was used as the effect size measure for individual effects. In addition to the variables mentioned above, a limited number of additional variables were assessed. Analyses pertaining to those variables are not described here for reasons of brevity.

### Results

#### Perceived appropriateness

The analyses for perceived appropriateness revealed a significant effect of the anger manipulation on perceived appropriateness, *t*(93) = 3.83, *p* < .001, *g* = 0.77, with lower levels of perceived appropriateness in the anger condition versus the non-emotional disagreement condition (see Table 4).

**Table 4:** Means and standard deviations of outcome measures in Study 4

	Anger (n = 48)		Disagreement (n = 48)	
	M	SD	M	SD
Perceived appropriateness	4.52^a^	0.89	5.16^b^	0.76
Persuasion	3.71	0.97	3.49	1.08
Perceived dominance	5.53^b^	0.79	5.17^a^	0.78
Conferred status	4.72	0.87	4.67	0.90

Different superscripts within a row indicate significant differences between conditions at *p* < .05

#### Persuasion

There was no significant main effect of the anger manipulation on persuasion, t(94) = − 1.07, *p* = .29, *g* = 0.21. The means did not show the expected pattern, with persuasion highest in the anger condition and lowest in the disagreement condition.

#### Perceived dominance and status conferral

The analyses revealed that the anger manipulation affected participants’ perceptions of the sender’s perceived dominance, *t*(93) = − 2.24, *p* = .03, *g* = 0.45, with the angry sender being seen as more dominant than the disagreeing sender. Conferred status did not show a significant effect of the anger manipulation, *t*(93) = − 0.25, *p* = .80, *g* = 0.06.

#### Mediation

As perceived appropriateness and perceived dominance both differed significantly between the anger condition and the disagreement condition, both qualified as potential mediators. Therefore, we entered both as the independent variables, together with condition, in a linear regression analysis with persuasion as the dependent variable. The results showed a significant effect of perceived appropriateness on persuasion, *B* = 0.34, *t*(90) = 2.72, *p* < .01, but a non-significant effect of perceived dominance, *B* = 0.17, *t*(90) = 1.31, *p* = .20. This result was confirmed when using separate mediation analyses using PROCESS. Whereas the analyses yielded a significant indirect effect for perceived appropriateness, ab = − 0.24, bootstrapped CI [− 0.45 to − 0.09], κ^2^ = .11, no substantial mediation was found for perceived dominance, ab = 0.06, bootstrapped CI [− 0.01 to 0.21], κ^2^ = .03.

## General discussion

Anger is a prominent feature of media messages. It therefore makes sense to investigate the consequences of this phenomenon for persuasion. The present research found that expressing anger was perceived as less appropriate than expressing non-emotional disagreement. The results of Study 2–4 furthermore suggest that perceived appropriateness mediates a negative effect of anger expression on persuasion - although the evidence for mediation in Study 1 was weak. In addition, anger expression lead to perceptions of the sender as low in likability and competence in Study 1 and 2. As such, it seems that in the context of mediated messages, anger expression is not a successful persuasive strategy.

So do these results lend support for the notion of a veritable backlash effect of anger expression? The total effect of anger expression on persuasion was neither significant nor strong in all four studies. The conclusion of a backlash effect would perhaps be too strong, therefore. Notably, however, the present research constitutes the first instance in which anger expression is contrasted with non-emotional expression of disagreement as a means to assess the effect of anger expression while holding the valence of the information constant. In this first test, we did not find any evidence in favor of a positive persuasive effect. In fact, considering the consistent negative effects of anger expression on perceived appropriateness, and the negative effects for likability and competence in Study 1 and 2, it seems there is some ground to assume that anger expression is more likely to have negative rather than positive effects for persuasion. (Perceived likability and competence were also assessed in Study 4; the analyses revealed the same pattern of results as in Study 1 and 2).

In the parlance of EASI theory (Van Kleef et al. 2011), the present research showed evidence of an affective, rather than an inferential process of anger expression. These results are in line with research on ‘incivility’ in political communication. This research has shown that political messages containing incivility are seen as less persuasive and often reflect negatively on the sender (Brooks and Geer 2007; Fridkin and Kenney 2008, 2011; Mutz and Reeves 2005). As for the mechanism underlying this effect, Mutz and Reeves (2005, p. 1) hypothesize that uncivil political messages “violate well-established social norms for the polite […] expression of opposing views.” The results of the present research seem to bear this out, showing that anger expression can result in lower levels of perceived appropriateness, and that perceived appropriateness in turn can affect persuasion. This finding is important, as we know of no previous direct empirical test of the mediating role of perceived appropriateness.

As such, we propose that the persuasive power of emotions may be more easily found for expressions of emotions such as happiness and sadness than for expressions of anger. The former may be subject to less stringent social norms than the latter (Geddes and Callister 2007). In other words, accepted social norms for polite conversation and discourse may act as a boundary condition for the persuasive power of anger expression.

So is it likely that anger expression will always backfire as a persuasive strategy? While Senator Sanders lost the primaries of the Democratic Party, Donald Trump went on to win the US 2016 presidential election. But in many cases, he may have won over voters *despite* his anger rather than because of it. It is well documented that many voters were put off by his angry personal style (Balz and Clement 2016). It is also notable that his failure to abide by socially acceptable ‘display rules’ for anger have led his opponents to paint pictures of him as emotionally unstable (Hillyard 2016). Still, anger expression may have worked for some voters. Future research should investigate the circumstances under which anger expression has positive persuasive effects. One factor that future research should look into is receivers’ feelings of antagonism and mistrust.

It is notable that our messages considered the policy proposals in terms of a rational discussion of their likely consequences. As such they employed what is known as an ‘issue frame’ (Brooks and Geer 2007). However, an issue can also be framed in terms of antagonism between groups or mistrust, for instance when it is discussed in terms of a perceived struggle between selfish politicians versus the common people, or between ‘Wall street’ and ‘Main Street’ (e.g., Barofsky 2012). Frijda (2007) describes anger as arising from ‘appraisals of offense’, which occur when others willfully harm our concerns. When recipients can be persuaded that there has been a willful harm of concerns, the usual social norms for the expression of opposing views (Mutz and Reeves 2005) may not be salient any longer and anger may seem the appropriate reaction instead.

One limitation of the present research is the correlational nature of the link between perceived appropriateness and persuasion. In our studies, perceived appropriateness was assessed by means of self-report, rather than manipulated experimentally. This leaves the ultimate question of causality unaddressed. Future research should manipulate perceived appropriateness rather than measure it. However, given people’s tendency to judge and punish those who do not conform to social norms (Chekroun and Brauer 2002), and previous studies finding negative effects of ‘offensive’ advertising (Beard 2008; Prendergast et al. 2008), we feel that our findings warrant the tentative conclusion that perceived inappropriateness hinders persuasion.

Other limitations of the present research include the use of undergraduate students as participants, the failure to assess long-term persuasive outcomes, and the use of a self-constructed anger measure in Study 3. Apart from focusing on perceptions of antagonism as moderators of the anger expression effect, a more diverse sample and assessment of long-term effects would go a long way to shine further light on the persuasive effects of anger expression. Furthermore, future research may want to replicate our findings using non-verbal (i.e., visual) manipulations of anger.

A major strength of our work, on the other hand, was the fact that we contrasted anger expression with non-emotional disagreement. In the first available research using such a test, we did not find evidence that anger expression can be persuasive. Rather, negative effects were found for perceived appropriateness and perceptions of the sender as likable and competent. Together, these results suggest that expressing anger as a persuasive strategy is a risky course of action.
